# Clinicopathologic and Molecular Features of Colorectal Adenocarcinoma with Signet-Ring Cell Component

**DOI:** 10.1371/journal.pone.0156659

**Published:** 2016-06-14

**Authors:** Qing Wei, Xicheng Wang, Jing Gao, Jian Li, Jie Li, Changsong Qi, Yanyan Li, Zhongwu Li, Lin Shen

**Affiliations:** 1 Department of Gastrointestinal Oncology, Key laboratory of Carcinogenesis and Translational Research (Ministry of Education), Peking University Cancer Hospital & Institute, Beijing, China; 2 Department of Pathology, Key Laboratory of Carcinogenesis and Translational Research (Ministry of Education), Peking University Cancer Hospital & Institute, Beijing, China; Institute of Basic Medical Sciences, Chinese Academy of Medical Sciences, CHINA

## Abstract

**Background:**

We performed a retrospective study to assess the clinicopathological characters, molecular alterations and multigene mutation profiles in colorectal cancer patients with signet-ring cell component.

**Methods:**

Between November 2008 and January 2015, 61 consecutive primary colorectal carcinomas with signet-ring cell component were available for pathological confirmation. RAS/BRAF status was performed by direct sequencing. 14 genes associated with hereditary cancer syndromes were analyzed by targeted gene sequencing.

**Results:**

A slight male predominance was detected in these patients (59.0%). Colorectal carcinomas with signet-ring cell component were well distributed along the large intestine. A frequently higher TNM stage at the time of diagnosis was observed, compared with the conventional adenocarcinoma. Family history of malignant tumor was remarkable with 49.2% in 61 cases. The median OS time of stage IV patients in our study was 14 months. RAS mutations were detected in 22.2% (12/54) cases with KRAS mutations in 16.7% (9/54) cases and Nras mutations in 5.4%(3/54) cases. BRAF V600E mutation was detected in 3.7% (2/54) cases. As an exploration, we analyzed 14 genes by targeted gene sequencing. These genes were selected based on their biological role in association with hereditary cancer syndromes. 79.6% cases carried at least one pathogenic mutation. Finally, the patients were classified by the percentage of signet-ring cell. 39 (63.9%) cases were composed of ≥50% signet-ring cells; 22 (36.1%) cases were composed of <50% signet-ring cells. We compared clinical parameters, molecular and genetic alterations between the two groups and found no significant differences.

**Conclusions:**

Colorectal adenocarcinoma with signet-ring cell component is characterized by advanced stage at diagnosis with remarkable family history of malignant tumor. It is likely a negative prognostic factor and tends to affect male patients with low rates of RAS /BRAF mutation. Colorectal patients with any component of signet-ring cells, regardless of the extent, shared similar clinicopathological characteristics, molecular alterations and genetic profiles.

## Introduction

The most recent estimates of the worldwide burden of cancer (GLOBOCAN 2012) show that colorectal cancer (CRC) is the third most commonly diagnosed cancer (1.36 million cases; 9.7%), and the fourth highest cause of cancer death (694,000 deaths; 8.5%) [1]. Three major histological subtypes of CRC can be identified: intestinal type adenocarcinoma, mucinous adenocarcinoma (MAC) and signet-ring cell carcinoma (SRCC). While typical adenocarcinomas are the most common tumors of the colon and rectum, the other two pathological subtypes have been reported to be associated with varied survival outcomes. The MAC and SRCC represent approximately 5–15% and 1% of the disease, respectively [2]. Because of their relatively rare occurrences, most studies on signet-ring cell colorectal carcinoma include both MAC and SRCC [3]. Fewer data exist regarding signet-ring cell subtype only. The World Health Organization (WHO) defines that signet-ring cell carcinoma is composed of ≥50% of tumor cells with prominent intracytoplasmic mucin, typically displacing and indenting the nucleus [4]. Clinicopathologic features and poor overall survival of SRCC have been suggested in recent studies [5,6]. However, colorectal adenocarcinoma with component of signet-ring cells less than 50% have been poorly understood, especially in the Asian population.

A number of genes and pathways have been implicated in colorectal carcinogenesis, and molecular alterations may be associated with different morphologic types of carcinomas. Ogino et al [7] demonstrated that BRAF mutation was more frequent in the signet-ring cell colorectal carcinoma whereas more frequent KRAS mutation was observed in the mucinous group. The association between RAS/BRAF status and pathological subtypes is still inconclusive.

Nearly 5% of all colorectal cancers (CRCs) are diagnosed in individuals who have a hereditary cancer syndrome [8]. In particular, signet-ring cell CRC has been more frequently observed in patients with hereditary non-polyposis colorectal cancer[9]. Additionally, hereditary diffuse gastric cancer demonstrated a unique signet-ring cell histotype under microscope [10]. Therefore, we explore if there were any associations between CRC with signet-ring cell component and hereditary gastrointestinal cancer syndromes. Advances in next-generation sequencing (NGS) technology have enabled us to simultaneously analyze multiple genes with high sensitivity and accuracy. In this study, we selected a panel of 14 genes related to hereditary cancer syndromes [11–19] and used the NGS to exam the genetic profile in CRC with signet-ring cell component.

The objectives of our study are to (1) assess the clinicopathological characters in CRC with signet-ring cell component; (2) correlate the histopathologic findings with molecular alterations, in particular, RAS and BRAF genes; (3)detect mutation frequencies in genes correlated with hereditary cancer syndromes.

## Methods and Materials

### Patient population

**Fig 1:**
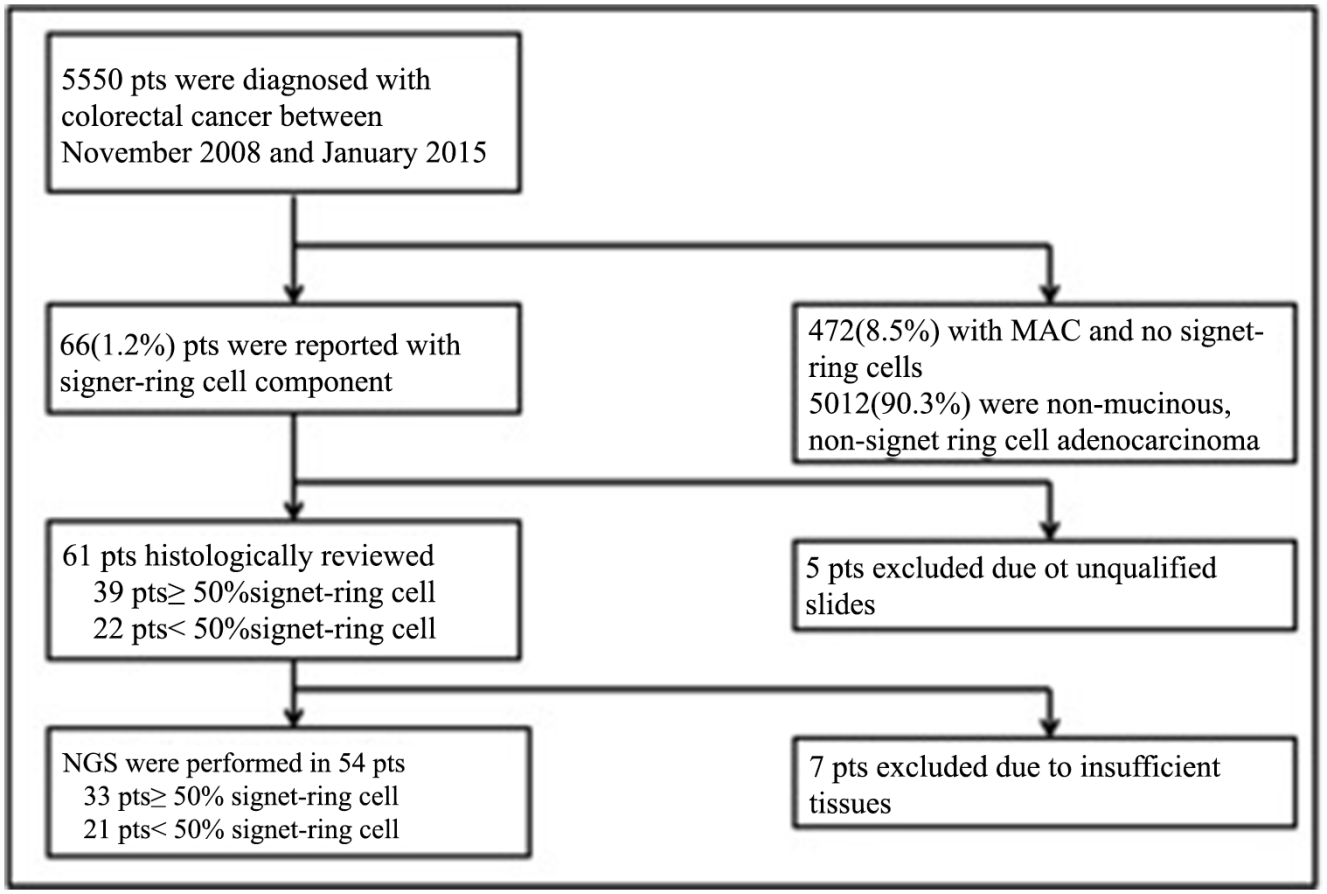
Work Flow Chart. pts = patients; NGS = next-generation sequencing; MAC = mucinous adenocarcinoma.

A total of 5550 primary colorectal carcinomas were surgically resected in Beijing Cancer Hospital from November 2008 to January 2015. Of these 5550 patients, 472(8.5%) cases were MAC without any signet-ring cell. 66(1.2%) cases contained signet-ring cell component. The rest of 5012 (90.3%) cases were diagnosed as non-mucinous, non-signet ring cell adenocarcinoma. 66 consecutive primary colorectal carcinomas with signet-ring cell component were further analyzed. Clinical parameters, including age, gender, date of surgery, tumor size, and tumor anatomic location at the initial presentation, family history of malignant tumor were obtained by reviewing the medical records. The tumor sites were classified as right-sided colon (ileocecal junction, cecum, ascending colon, hepatic flexure and transverse colon), left-sided colon (splenic flexure, descending colon and sigmoid colon) or rectum. All tumors were staged according to the TNM staging system of the American Joint Committee on Cancer (7th version, 2009). In this study, patients ≤40 yrs at diagnosis were referred to as young patients. Intraoperative and clinical follow-up data were obtained from hospital and clinic charts. The date of last follow-up was August 2015. The main survival index was overall survival (OS). All patients gave the written informed consents for their samples to be used in medical research. This study was approved by the Ethics Committee of Peking University Cancer Hospital and performed according to the Declaration of Helsinki Principles. The study flow chart was illustrated in Fig 1.

### Pathologic Evaluation

Of these 66 patients, 61 were histologically reviewed to confirm the percentage of signet-ring cells. Hematoxylin and eosin-stained slides of the tumors were reviewed by pathologists. Tumors were classified according to the amount of their signet-ring cell component: the cutoff value was 50%. Group A was defined as a lesion consisting of ≥50% signet-ring cells while Group B was defined as a lesion consisting of < 50% signet-ring cells. For patients who have received neoadjuvant therapy, the pretreatment biopsy samples were also reviewed.

### KRAS, NRAS and BRAF Mutation Analysis

Genomic DNA of formalin-fixed, paraffin-embedded (FFPE) sections with ≥50% tumor cells (if the content of tumor cells in sections was lower than 50%, the sections would be microdissected) was extracted using E.Z.N.A.FFPE DNA Kit (Lot. D3399-01,OMEGA, USA) according to the manufacturer’s instructions. All genomic DNAs were stored at −20°C until further research. DNA fragments including KRAS/NRAS gene(exon 2/3/4) were amplified by PCR using primers, as with the DNA fragment including exon 15 of BRAF gene. Each PCR reaction consisted of 10× LA PCR buffer II 2 μl, 2.5 mmol/L dNTPs 2 μl, LA Taq 0.1 μl (DRR200A, TAKARA), genomic DNA 2 μl, 10 μmol/L forward primer 0.5 μl, and 10 μmol/L reverse primer 0.5 μl in a final volume of 20 μl. The cycling conditions were 95°C for 5 min, 45 cycles of 95°C for 30 s, 56°C for 45 s and 72°C for 20 s, final extension at 72°C for 5 min. The detailed sequencing procedures have been published[20].

### Multigene sequencing by NGS

54 patients with FFPE tissues were performed the Next-Generation Sequencing. Genomic DNAs were extracted from paraffin-embedded tumor specimens using QIAamp DNA FFPE Tissue Kit (Qiagen, Hilden, Germany). Three microgram (3 μg) genomic DNA was used for library preparation according to the manufacturer’s instruction (MyGenostics, Beijing, China), and the final library size 350–450 bp containing adapter sequences was used in the following experiment. A panel of 14 genes related to hereditary syndrome was captured using the OncoCap Enrichment System (MyGenostics, Beijing, China). After enrichment, the enriched libraries were sequenced on an Illumina Solexa HiSeq 2000 sequencer for paired read 100 bp followed by data retrieved using Solexa QA package and cutadapt program (http://code.google.com/p/cutadapt/). Short read mapping and alignment were performed using BWA software (Burrows Wheeler Aligner). SNPs and indels were detected using the SOAPsnp software and GATK Indel Genotyper (http://www.broadinstitute.org/gsa/wiki/index.php/; The Genome Analysis Toolkit), respectively. All reference sequences were based on the NCBI37/hg19 assembly of the human genome. The mutation profile was drawn using R software after filtering data based on following references: the coverage of mutated allele ≥5; mutation ratio ≥0.1; absence of mutation in 1000 Genomes Project, and non-synonymous mutations.

### Variants Classification

Sequence variants and large insertions and deletions were classified according to the American College of Medical Genetics (ACMG) guidelines for variant interpretation [21]. Deleterious mutations included nonsense and frameshift mutations predicted to result in protein truncation, as well as specific missense and intronic alterations that had been recognized previously as deleterious based on supporting linkage, functional, biochemical, and/or statistical evidence. ANNOVAR package was used to predict their functional consequences such as silent or nonsilent variants for somatic variants located in coding sequences[22]. Suspected deleterious mutations included alterations for which available evidence indicated a high likelihood, but not confirmation, of pathogenicity. Individuals with deleterious or suspected deleterious genomic alterations were defined collectively as having pathogenic mutations. Alterations were deemed benign if available evidence indicated a low likelihood that such alterations altered normal gene expression and/or function. Alterations were classified as variants of uncertain significance (VUS) if data was insufficient to support either a deleterious or benign interpretation.

### Statistical Analysis

Statistical analyses were performed with SPSS 13.0 software. Chi-square test or Fisher’s exact test was used when comparing frequencies between groups. Differences between means of groups were compared by independent sample T-test. The period from the date of resection to the date of death or last contact (if alive) was used for survival analysis. The log-rank test was used to compare Kaplan–Meier survival curves. All tests were two-sided and P-value less than 0.05 was considered statistically significant.

## Results

### Clinicopathological Characteristics

A total of 61 patients with primary colorectal signet-ring cell carcinomas (age 25–83 yrs, median age 54.9yrs) were evaluated, including 36(59%) male and 25(41%) female patients. At diagnosis, 43 (70.5%) patients were older than 40 yrs, whereas only 18(29.5%) were considered young patients as defined in this study. As for the tumor location, 25(41.0%) tumors were located on right-sided colon, 23(37.7%) tumors were located on rectum, while 13(21.3%) tumors were located on left-sided colon. The majority of patients exhibited III stage (62.3%), whereas minority of patients (11.5%) exhibited I&II stages. Family cancer history could be identified in 30 (49.2%) patients. In particular, 15(24.6%) of them had a family history of gastric or colon cancer. The clinicopathologic features of colorectal carcinoma with signet-ring cell component are detailed in Table 1.

**Table 1:** Clinicopathologic features of colorectal adenocarcinoma with signet-ring cell component.

N*	61
**Sex**	
** male**	36(59.0%)
** female**	25(41.0%)
**Age**	
**>40yr**	43(70.5%)
**≤40yr**	18(29.5%)
**Mean median age**	54.9 yr
**Location**	
**left**	13(21.3%)
**right**	25(41.0%)
**rectum**	23(37.7%)
**Stage**	
**I-II**	7(11.5%)
**III**	38(62.3%)
**IV**	16(26.2%)
**Family History of Malignancy**	
**Yes**	30(49.2%)
**No**	31(50.8%)

N* = the number of patients

### KRAS, NRAS and BRAF Mutation Analysis

RAS mutations could be detected in 22.2% cases with KRAS mutations in 16.7% (9/54) cases, and NRAS mutations in 5.6%(3/54) cases (Table 2). The most common KRAS mutations occurred at codons 12 (33.3%, 3/9) and 13 (22.2%, 2/9) of exon 2, while 2 cases occurred in exon 3 and the rest of 2 cases occurred in exon 4. Two cases of NRAS mutations in exon 3(codons 61) and one case in exon 4 (codons 117) were detected. BRAF mutations were identified in 3.7% (2/54) cases. RAS and BRAF mutations were mutually exclusive.

**Table 2:** KRAS, NRAS and BRAF status Analysis.

N^1^	54
Ras/Braf WT^2^	40(74.1%)
Ras Mut^3^	12(22.2%)
Braf Mut	2(3.7%)

^1.^ N = the number of patients

^2.^ WT = wild type

^3.^ Mut = mutation

### Multigene Mutation Profiling by NGS

We also analyzed the distributions of pathogenic variants in 14 genes. These genes included MLH1, MSH2, MSH6, PMS2, CDH1, APC, MUTYH, STK11, SMAD4, PTEN, BMPR1A, BRCA1/2, and EPCAM which were well studied in various hereditary cancer syndromes.79.6% cases (43/54) carried at least one pathogenic mutation in 13 genes (Table 3), and pathogenic mutation in EPCAM was not detected. Except PMS2 and PTEN, a total of 98 VUS were identified in the other 12 genes among 31 patients. Per gene, the median number of VUS identified across all 54 participants was 5, ranging from 0 (PTEN, PMS2) to 20 (BRCA2).

**Table 3:** Multigene Mutation Profiling by NGS.

	Total(%)
N*	54
**mutated N**	43 (79.6)
**APC**	17 (31.5)
**BMPR1A**	8 (14.8)
**BRCA1**	14 (25.9)
**BRCA2**	9 (16.7)
**CDH1**	13 (24.1)
**EPCAM**	0 (0)
**MLH1**	9 (16.7)
**MSH2**	7 (13.0)
**MSH6**	11 (20.4)
**PMS2**	2 (3.7)
**MUYTH**	5 (9.3)
**PTEN**	4 (7.4)
**SMAD4**	22 (40.7)
**STK11**	7 (13.0)

N* = the number of patients

By searching the Human Gene Mutation Database (HGMD), we identified 50 pathogenic mutations which had been reported in hereditary cancer syndromes associated germline mutations in published literatures (S1 Table).

### Clinical Outcome of CRC with signet-ring cell component

Overall survival as a clinical outcome index was assessed. Follow-up status was known in 61 patients with a median follow-up time of 20.5 months (range:3 to 60 mos). The median OS time of all the patients was 35.7 months. For stage IV patients, the median OS was 14 months, while the median OS of stage III patients was 35.9 months. As anticipated, patients who were in earlier stage and received radical surgery survived longer.

### Comparisons of Clinical Parameters, Molecular Markers and Genetic Profiles between Group A and Group B

To explore the influence of signet-ring cell on clinical, molecular and genetic variables, we divided 54 patients with sufficient tumor samples into two groups. For Group A patients with ≥50% signet-ring cell component, the mean age was 51.7yrs (range: 25–77 yrs) with a slight male predominance (56.4%), although this was not statistically significant compared with Group B patients (signet-ring cell component <50%) (*P = 0*.*582*). In Group A, nearly half of the cases (17/39, 43.6%) were identified in the right-sided colon while Group B patients tended to present mostly in the rectum (*P = 0*.*392*). No difference was identified between the two groups with regard to tumor stage (*P = 0*.*56*). Obviously, stage I-II diseases were rare in both groups. Moreover, both groups presented with a high rate of family cancer history (46.2% vs 54.5%, p = 0.358) (Table 4).

**Table 4:** Clinicopathologic features of colorectal adenocarcinomas stratified by signet-ring cell component.

	Group A		Group B	
**Clinicopathologic features**				
**N**^1^	39		22	
**Sex**				
**male**	22 (56.4%)		14 (63.6%)	
**female**	17 (43.6%)		8 (36.4%)	*p = 0*.*582*
**Age**				
**>40yr**	26(66.7%)		17(77.3%)	
**≤40yr**	13(33.3%)		5(22.7%)	
**Mean median age**	51.7yr		60.7yr	*P = 0*.*284*
**Location**				
**left**	9 (23.1%)		4(18.2%)	
**right**	17(43.6%)		8(36.4%)	
**rectum**	13(33.3%)		10(45.5%)	*P = 0*.*645*
**Stage**				
**I-II**	4(10.3%)		3(13.6%)	
**III**	25(64.1%)		13(59.1%)	
**IV**	10(25.6%)		6(27.3%)	*p = 0*.*9*
**Family History of Malignancy**				
**Yes**	18 (46.2%)		12 (54.5%)	
**No**	21 (53.8%)		10 (45.5%)	*p = 0*.*358*
**Ras, and Braf Mutation**				
**N**^1^	33	21		
**Ras/Braf WT**^2^	27(81.8%)	13(61.9%)		
**Ras Mut**^3^	5(15.2%)	7(33.3%)		
**Braf Mut**^3^	1(3.0%)	1(4.8%)		

^1^N = the number of patients

^2^WT = wild type

^3^Mut = mutation

The Kaplan–Meier survivals were performed between the groups with the same stage. No statistically significant differences in overall survival were identified between Group A and Group B. Moreover, the median OS of Group A was slightly longer than Group B (38.4 months *vs* 31.5 months, *p = 0*.*235*). The median OS of stage IV in Group A and Group B were 18.3 months and 13.6 months, respectively (*P = 0*.*070*).

Group A displayed less frequent RAS mutations (5/33, 15.2%) in comparison to Group B (7/21, 33.3%)(*P = 0*.*218*). Each group harbored one case of BRAF V600E mutation (Table 4).

27 patients (81.8%) in Group A and 16 patients (76.2%) in Group B carried the pathogenic variants. The prevalence of mutations in APC, BMPR1A, BRCA1/2 and MSH6 were slightly higher in Group A, while mutations in CDH1, EPCAM, MLH1, MSH2, PMS2, MUYTH, PTEN, SMAD4, and STK11 were slightly higher in Group B (S2 Table). In Group A, 66 VUS were detected among 12 genes in 23 patients, whereas 32 VUS were found in 10 genes among 8 patients in Group B.

## Discussion

Earlier studies indicated that SRCC was more frequently found on right-sided colon and female dominated [6,23–25]. By contrast, our results show that colorectal cancer with signet-ring cell component could be frequently found in left-sided colon and rectum and with a slight male predominance. This was in line with recent report by Tan Y[26],which was also focused on Asian population. A more advanced TNM stage at the time of diagnosis, compared with the conventional adenocarcinomas, was confirmed by our study which was consistent with previous investigation [2,3,27–30]. It has been reported that about 30% CRC cases were linked with family aggregation [31]. We found that family history of malignant tumor in our study patients were remarkable with 49.2% (30/61). The median age was 54.9yrs in our study, while 70% of patients diagnosed with CRC were older than 65 yrs in general population [32]. It is worth mentioning that colorectal cancer patients with signet-ring cell component might exhibit early-onset tendency.

Signet-ring cell carcinoma is a rare histologic subtype of colorectal cancer with a poor prognosis [2,3,27–30]. Our findings revealed that the median OS time of stage IV patients in our study was 14 months, which was much shorter than the 23.4 months in regular adenocarcinomas with the same stage [33]. There was no significant difference in overall survivals between Group A and Group B patients, which was consistent with the result of a previous report [26]. This similarity between the two groups has a critical clinical meaning. Based on our results, we speculate the existence of signet-ring cells may be a negatively prognostic maker regardless the percentage of signet-ring cell component.

The previous literature contained limited information on the molecular features of colorectal signet-ring cell carcinoma. Ogino et al[7] examined BRAF mutation and KRAS mutation in colorectal carcinoma with signet-ring cell component, and did not identify KRAS mutation in any of the 8 signet-ring cell carcinoma cases, all mutations were seen in 33% of cases that had <50% signet-ring cells. The average KRAS mutation rate was 26% in colorectal cancer with varied signet-ring cell component. Our results identified a lower rate of KRAS mutation (16.7%), and the whole RAS mutation rate was 22.2%. Group B harbored a higher RAS mutation rate with 33.3% which was in line with Ogino’s study that most of the KRAS mutations were detected in <50 signet-ring cell group (33.3% vs 15.2%, *P = 0*.*296*). However, Sanjay Kakar et al[33] reported that KRAS mutations in codons 12 and 13 were observed in half of signet-ring cell carcinomas, which was similar to the rate in conventional adenocarcinomas. The reason for this discrepancy is not entirely clear. Only two cases (3.7%) of BRAF V600E mutation were identified in all the tumors with one case in each group, which was considerably lower than studies reported by Ogino et al [7]and Sanjay Kakar et al[34]. Their studies showed that 22% and 33% BRAF mutation in signet-ring cell carcinomas, respectively. Ogino et al also reported that the average BRAF mutation rate was 28% in colorectal cancer with varied signet-ring cell component. It has been suggested that BRAF V600E mutation was associated with hypermythylation in MLH1 promoter region [35]. The epigenetic changes of CRC with signet-ring cell component in Chinese patients might differ from other ethnic populations. Further study on this aspect is deserved.

As mentioned earlier, the high incidence of family malignancy history and early-onset tendency in CRC with signet-ring cell component led us to investigate if this unique pathological subtype has a correlation with hereditary cancer syndromes. MLH1, MSH2, MSH6, PMS2, CDH1,APC, MUTYH,STK11,SMAD4,PTEN, BMPR1A, BRCA1/2, and EPCAM are well studied genes in hereditary cancer syndromes, such as Lynch syndrome, hereditary diffuse gastric cancer and etc. In present study, we also looked into the above gene status in our cases by NGS approach. Though no significant difference was identified in the pathogenic mutations between Group A and Group B, we identified that 79.6% cases carried at least one pathogenic mutation in one of above 13 genes, except EPCAM. In particular, 50 pathogenic mutations revealed by NGS sequencing had been reported in hereditary cancer associated germline mutations in early studies. Since CRC patients with signet-ring cell component had a strong family history of malignancy (nearly 50%), in combination with the above genetic mutations, we might be able to determine potential candidate genes for germline test. It would be a first attempt to establish a link between signet-ring cell CRC and hereditary cancer syndromes in Chinese population.

Recently, the CRC Subtyping Consortium (CRCSC) divided CRC into four consensus molecular subtypes (CMSs) with distinguishing features: CMS1 (microsatellite instability immune, 14%); CMS2 (canonical, 37%); CMS3 (metabolic, 13%); and CMS4 (mesenchymal, 23%)[36]. Samples with mixed features (13%) possibly represent a transitional phenotype or intratumoral heterogeneity. Notably, the CMS1 population with relatively high MSI and BRAF mutation rate has a very poor survival rate after relapse. Signet-ring cell colorectal cancer was considered as this category due to harboring higher frequencies of BRAF mutation and high levels of microsatellite instability (MSI-H) [7]. However, our findings in CRC with signet-ring cell component indicated that this peculiar subtype might not fit in any molecular subgroup. In regard to this dilemma, whole genome study in CRC with signet-ring cell component is warranted.

To our knowledge, this study was the first to analyze clinical parameters, molecular and genetic alterations among CRC patients with signet-ring cell component. However, our study had some limitations. First, our study was retrospective and conducted in a single academic center with limited sample size. Second, data on multigene analysis in hereditary disease is still preliminary. Proper interpretation of NGS data and the selection of gene(s) for germline testing require further investigation.

In conclusion, our study showed that colorectal cancer with signet-ring cell component was presented with male predominance and early onset tendency, equally distributed among left-sided colon, right-sided colon and rectum, associated with poor prognosis. And this unique morphological subtype was correlated with low rates of RAS and BRAF mutations. Colorectal cancer with signet-ring cell, regardless of the extent, shared similar clinicopathological characteristics and molecular and genetic alterations. In addition, considering high prevalence of family tumor history and pathogenic mutations in hereditary related genes, family history details and germline testing should be arranged in this population.

## Supporting Information

S1 File Clinical studies checklist. (DOCX)

S2 File Study protocol. (DOCX)

S3 File Next-generation sequencing result. (ZIP)

S1 Table Pathogenic mutations found in patients’ tumors by NGS and their nucleotide changes associated with germline mutations reported from literatures. (DOCX)

S2 Table Multigene Mutation Profiling by NGS. N = the number of patients.
(DOCX)
