# Supplementary material for: Clinicopathologic and Molecular Features of Colorectal Adenocarcinoma with Signet-Ring Cell Component
Source: PLoS One. 2016 Jun 14;11(6):e0156659. doi: 10.1371/journal.pone.0156659 (PMC4907485; doi:10.1371/journal.pone.0156659)
Supplement: S2 File — (DOCX) [file pone.0156659.s002.docx]

**Clinicopathologic and molecular features of colorectal adenocarcinoma with signet-ring**

**cell component**

**Study protocol**

| **Sponsor** | **Peking University Cancer Hospital & Institute** |
| --- | --- |
| **Investigators** | **Lin Shen MD.PHD and ZhongWu Li MD.PHD** |
| **Version Number** | **Version 1.0** |
| **Version Date** | **2014.12.** |

**The study protocol**

| Official scientific title | Clinicopathologic and molecular features of colorectal adenocarcinoma with signet-ring cell component |
| --- | --- |
| Sponsor | Peking University Cancer Hospital & Institute |
| Investigators | Lin Shen MD.PHD；ZhongWu Li MD.PHD |
| Brief summary | Signet-ring cell carcinoma (SRCC) of colorectal cancer represents approximately 1% of the disease. Because of their relatively rare occurrence, most studies on signet-ring cell colorectal carcinoma include both SRCC and other pathological types. We aim to perform a retrospective study to assess the clinicopathological characters，molecular alterations and multigene mutation profile in colorectal cancer patients with signet-ring cell component. |
| Study type | Observational |
| Study design | Time perspective: retrospective |
| Sampling method | Non-probability sample |
| Study population | Colorectal cancer patients with signet-ring cell component in the tumor tissue |
| Condition | Colorectal cancers |
| Intervention | Laboratory biomarker analysis  Retrospective data collection |
| Eligibility criteria | Inclusion criteria:   - Adult patients >=18 years of age - Primary colorectal carcinomas with signet-ring cell component   Exclusion criteria:   - Tissue samples were not available for confirmation and analysis |
| Gender | Both |
| Ages | 18 years and older |
| Accepts healthy volunteers | No |
| Anticipate start date | Feburary,2015 |
| Estimated enrollment | 70 |
| Estimated completion date | December,2015 |
| Contacts | Lin Shen 010-88196561 [lin100@medmail.com.cn](mailto:lin100@medmail.com.cn);  ZhongWu Li 010-88196561 [zhwuli@hotmail.com](mailto:zhwuli@hotmail.com) |
