# Supplementary material for: Clinicopathologic and Molecular Features of Colorectal Adenocarcinoma with Signet-Ring Cell Component
Source: PLoS One. 2016 Jun 14;11(6):e0156659. doi: 10.1371/journal.pone.0156659 (PMC4907485; doi:10.1371/journal.pone.0156659)
Supplement: S1 Table — (DOCX) [file pone.0156659.s004.docx]

**S1** Table. Pathogenic mutations found in patients’ tumors by NGS and their nucleotide changes associated with germline mutations reported from literatures.

| Genes | EXON | Nucleotide change | Assumed consequence | Disease | References |
| --- | --- | --- | --- | --- | --- |
| APC | exon16 | c.4588G>T | p.E1530X | Adenomatous polyposis coli | Bisgaard,et al ^1^ |
|  | exon14 | c.1660C>T | p.R554X | Adenomatous polyposis coli | Fodde,et al ^2^ |
|  | exon16 | c.2626C>T | p.R876X | Adenomatous polyposis coli | Miyaki,et al ^3^ |
|  | exon16 | c.3709C>A | p.Q1237K | Adenomatous polyposis coli | Jang,et al ^4^ |
|  | exon8 | c.710C>G | P.S237C | Adenomatous polyposis coli | Lopez-Kostner,et al ^5^ |
|  | exon8 | c.646C>T | P.R216X | Adenomatous polyposis coli | Lamlum,et al ^6^ |
|  | exon16 | c.3340C>T | p.R1114X | Adenomatous polyposis coli | Nagase,et al ^7^ |
|  | exon6 | c.637C>T | p.R213X | Adenomatous polyposis coli | Miyoshi,et al ^8^ |
| BMPR1A | - | - | - | - | - |
| BRCA1 | exon10 | c.3710T>A | p.I1237K | Breast and/or ovarian cancer | Johannsson,et al ^9^ |
|  | exon20 | c.5194-2A>T |  | Breast and/or ovarian cancer | Robertson,et al ^10^ |
|  | exon19 | c.5252G>A | p.R1751Q | Breast and/or ovarian cancer | Gad,et al ^11^ |
|  | exon13 | c.6952C>T | p.R2318X | Breast and/or ovarian cancer | Zhou,et al ^12^ |
|  | exon10 | c.2083G>A | p.D695N | Breast and/or ovarian cancer | Greenman,et al ^13^ |
|  | exon10 | c.1945G>A | p.E649K | Breast and/or ovarian cancer | Li,et al ^14^ |
|  | exon4 | c.203T>A | p.I68K | Breast and/or ovarian cancer | Abkevich,et al ^15^ |
|  | exon10 | c.800C>T | p.S267L | Breast and/or ovarian cancer | Caux-Moncoutier V,et al ^16^ |
|  | exon6 | c.427G>A | p.E143K | Breast and/or ovarian cancer | Shattuck-Eidens,et al ^17^ |
|  | exon23 | c.5506G>A | p.E1836K | Breast and/or ovarian cancer | Diez-Gilbert,et al ^18^ |
|  | exon10 | c.2591C>A | p.S864X | Breast and/or ovarian cancer | Marroni,et al ^19^ |
|  | exon10 | c.1487G>A | p.R496H | Breast and/or ovarian cancer | Schoumacher,et al ^20^ |
|  | exon19 | c.5246C>T | p.P1749L | Breast and/or ovarian cancer | Gayther,et al ^21^ |
| BRCA2 | exon13 | c.6952C>T | p.R2318X | Breast and/or ovarian cancer | Wagner,et al ^22^ |
|  | exon24 | c.9154C>T | p.R3052W | Breast and/or ovarian cancer | Kuznetsov,et al ^23^ |
|  | exon11 | c.6281A>T | p.Y2094F | Breast and/or ovarian cancer | Jakubowska,et al ^24^ |
|  | exon9 | c.742G>A | p.A248T | Breast and/or ovarian cancer | Fackenthal,et al ^25^ |
|  | exon23 | c.9007G>T | p.G3003X | Breast and/or ovarian cancer | Zhang,et al ^26^ |
|  | exon7 | c.520C>T | p.R174C | Breast and/or ovarian cancer | [Théry JC](http://www.ncbi.nlm.nih.gov/pubmed/?term=Th%C3%A9ry%20JC%5BAuthor%5D&cauthor=true&cauthor_uid=21673748),et al ^27^ |
|  | exon10 | c.1804G>A | p.G602R | Breast and/or ovarian cancer | Michils,et al ^28^ |
|  | exon11 | c.6235G>T | p.V2079L | Breast and/or ovarian cancer | Lecarpentier,et al ^29^ |
|  | exon18 | c.8219T>A | p.L2740X | Breast and/or ovarian cancer | Popp,et al ^30^ |
|  | exon17 | c.7863T>A | p.Y2621X | Breast and/or ovarian cancer | Esteban Cardeñosa E,et al ^31^ |
| CDH1 | exon12 | c.1901C>T | p.A634V | Gastric cancer | Suriano,et al ^32^ |
| EPCAM | - | - | - | - | - |
| MLH1 | exon13 | c.1459C>T | p.R487X | Colorectal cancer | Fidalgo,et al ^33^ |
|  | exon9 | c.704A>T | p.D235V | Colorectal cancer | Hu,et al ^34^ |
|  | exon4 | c.318C>G | p.S106R | Colorectal cancer | Auclair,et al ^35^ |
|  | exon10 | c.884+2T>A | Splice defect | Colorectal cancer | Mueller-Koch,et al ^36^ |
|  | exon14 | c.1614G>A | p.W538X | Colorectal cancer | Yuan,et al ^37^ |
| MSH2 | exon6 | c.943-1G>C | Splice defect | Colorectal cancer | Mangold,et al ^38^ |
| MSH6 | exon4 | c.2983G>A | p.E995K | Colorectal cancer | Vahteristo,et al ^39^ |
|  | exon3 | c.604C>A | p.P202T | Colorectal cancer | [Giráldez MD](http://www.ncbi.nlm.nih.gov/pubmed/?term=Gir%C3%A1ldez%20MD%5BAuthor%5D&cauthor=true&cauthor_uid=20924129),et al ^40^ |
|  | exon5 | c.3425C>A | p.T1142K | Colorectal cancer | [Pérez-Cabornero L](http://www.ncbi.nlm.nih.gov/pubmed/?term=P%C3%A9rez-Cabornero%20L%5BAuthor%5D&cauthor=true&cauthor_uid=23523604),et al ^41^ |
|  | exon5 | c.3284G>A | p.R1095H | Colorectal cancer | Kariola,et al ^42^ |
| MUTYH | exon3 | c.271G>A | p.D91N | Colorectal cancer | Nielsen,et al ^43^ |
| PMS2 | - | - | - | - | - |
| PTEN | exon8 | c.821G>T | p.W274L | Developmental delay | McBride,et al ^44^ |
|  | Exon7 | c.697C>T | p.R233X | Cowden disease | Liaw,et al ^45^ |
| SMAD4 | exon9 | c.988G>T | p.E330X | Juvenile polyposis | Gallione,et al ^46^ |
|  | exon9 | c.1082G>A | p.R361H | Juvenile polyposis coli | Kim,et al ^47^ |
| SKT11 | exon4 | c.580G>A | p.D194N | Peutz-Jeghers syndrome | Westerman,et al ^48^ |
|  | exon8 | c.971C>T | p.P324L | Peutz-Jeghers syndrome | Yoon,et al ^49^ |
|  | exon3 | c.427G>A | p.V143M | Peutz-Jeghers syndrome | Lim,et al ^50^ |

[1] Bisgaard ML, Ripa RS, Bülow S. (2004)Mutation analysis of the adenomatous polyposis coli (APC) gene in Danish patients with familial adenomatous polyposis (FAP). Hum Mutat 23:522.

[2] Fodde R, van der Luijt R, Wijnen J, Tops C, van der Klift H,et al. (1992)Eight novel inactivating germ line mutations at the APC gene identified by denaturing gradient gel electrophoresis.Genomics 13:1162-1168.

[3] Miyaki M, Konishi M, Kikuchi-Yanoshita R, Enomoto M, Igari T,et al.(1994) Characteristics of somatic mutation of the adenomatous polyposis coli gene in colorectal tumors.Cancer Res 54:3011-3020.

[4] Jang YH, Lim SB, Kim MJ, Chung HJ, Yoo HW,et al.(2010) Three novel mutations of the APC gene in Korean patients with familial adenomatous polyposis. Cancer Genet Cytogenet 200:34-39.

[5] Lopez-Kostner F, Alvarez K, de la Fuente M, Wielandt AM, [Orellana P](http://www.ncbi.nlm.nih.gov/pubmed/?term=Orellana%20P%5BAuthor%5D&cauthor=true&cauthor_uid=21488302),et al.(2010) Novel human pathological mutations. Gene symbol: APC. Disease: adenomatous polyposis coli.Hum Genet 2010 127:480.

[6] Lamlum H, Ilyas M, Rowan A, Clark S, Johnson V,et al.(1999)The type of somatic mutation at APC in familial adenomatous polyposis is determined by the site of the germline mutation: a new facet to Knudson's 'two-hit' hypothesis. Nat Med 5:1071-1075.

[7] Nagase H, Miyoshi Y, Horii A, Aoki T, Petersen GM,et al. (1992) Screening for germ-line mutations in familial adenomatous polyposis patients: 61 new patients and a summary of 150 unrelated patients. Hum Mutat 1:467-473.

[8] Miyoshi Y, Ando H, Nagase H, Nishisho I, Horii A,et al. (1992)Germ-line mutations of the APC gene in 53 familial adenomatous polyposis patients.Proc Natl Acad Sci U S A 89:4452-4456.

[9] Johannsson O, Ostermeyer EA, Håkansson S, [Friedman LS](http://www.ncbi.nlm.nih.gov/pubmed/?term=Friedman%20LS%5BAuthor%5D&cauthor=true&cauthor_uid=8644702), [Johansson U](http://www.ncbi.nlm.nih.gov/pubmed/?term=Johansson%20U%5BAuthor%5D&cauthor=true&cauthor_uid=8644702),et al. (1996) Founding BRCA1 mutations in hereditary breast and ovarian cancer in southern Sweden. Am J Hum Genet 58:441-450.

[10] Robertson L, Hanson H, Seal S, Warren-Perry M, Hughes D,et al. (2012) BRCA1 testing should be offered to individuals with triple-negative breast cancer diagnosed below 50 years.Br J Cancer106:1234-1238.

[11] Gad S, Caux-Moncoutier V, Pagès-Berhouet S, Gauthier-Villars M, Coupier I,et al. (2002)Significant contribution of large BRCA1 gene rearrangements in 120 French breast and ovarian cancer families.Oncogene 21:6841-6847.

[12] Zhou YZ, Sun Q, Lin SQ, Wang J, Liu B,et al. (2004) Germline mutations in the BRCA1 and BRCA2 genes from breast cancer families in China Han people. Zhonghua Yi Xue Za Zhi 84:294-298.

[13] Greenman J, Mohammed S, Ellis D, Watts S, Scott G,et al.(1998) Identification of missense and truncating mutations in the BRCA1 gene in sporadic and familial breast and ovarian cancer. Genes Chromosomes Cancer 21:244-249.

[14] Li N, Zhang X, Cai Y, Xu X, Zhang L,et al. (2006) BRCA1 germline mutations in Chinese patients with hereditary breast and ovarian cancer. Int J Gynecol Cancer 1:172-178.

[15] Abkevich V, Zharkikh A, Deffenbaugh AM, Frank D, Chen Y,et al. (2004)Analysis of missense variation in human BRCA1 in the context of interspecific sequence variation. J Med Genet 41:492-507.

[16] Caux-Moncoutier V, Castéra L, Tirapo C, Michaux D, Rémon MA,et al. (2011) EMMA, a cost- and time-effective diagnostic method for simultaneous detection of point mutations and large-scale genomic rearrangements: application to BRCA1 and BRCA2 in 1,525 patients.Hum Mutat 32:325-334.

[17] Shattuck-Eidens D, Oliphant A, McClure M, McBride C, Gupte J,et al.(1997) BRCA1 sequence analysis in women at high risk for susceptibility mutations. Risk factor analysis and implications for genetic testing.JAMA 278:1242-1250.

[18] Díez Gibert O, del Río E, Domènech M, Hernández EM, Sanz J,et al. (1999) Mutations in the BRCA1 gene in young Spanish women with breast cancer. Med Clin (Barc) 112:51-54.

[19] Marroni F, Aretini P, D'Andrea E, Caligo MA, Cortesi L, et al. (2004) Penetrances of breast and ovarian cancer in a large series of families tested for BRCA1/2 mutations. Eur J Hum Genet 12:899-906.

[20] Schoumacher F, Glaus A, Mueller H, Eppenberger U, Bolliger B,et al. (2001)BRCA1/2 mutations in Swiss patients with familial or early-onset breast and ovarian cancer. Swiss Med Wkly131:223-226.

[21] Gayther SA, Harrington P, Russell P, Kharkevich G, Garkavtseva RF,et al. (1996)Rapid detection of regionally clustered germ-line BRCA1 mutations by multiplex heteroduplex analysis. UKCCCR Familial Ovarian Cancer Study Group.Am J Hum Genet. 58:451-456.

[22] Wagner T, Stoppa-Lyonnet D, Fleischmann E, Muhr D, Pagès S,et al. (1999) Denaturing high-performance liquid chromatography detects reliably BRCA1 and BRCA2 mutations.Genomics. 62:369-376.

[23] Kuznetsov SG, Liu P, Sharan SK.(2008) Mouse embryonic stem cell-based functional assay to evaluate mutations in BRCA2.Nat Med 14:875-881.

[24] Jakubowska A, Nej K, Huzarski T, Scott RJ, Lubiński J. (2002) BRCA2 gene mutations in families with aggregations of breast and stomach cancers.Br J Cancer 87:888-891.

[25] Fackenthal JD, Sveen L, Gao Q, Kohlmeir EK, Adebamowo C,et al. (2005) Complete allelic analysis of BRCA1 and BRCA2 variants in young Nigerian breast cancer patients. J Med Genet 42:276-281.

[26] Zhang J, Pei R, Pang Z, Ouyang T, Li J,et al.(2012)Prevalence and characterization of BRCA1 and BRCA2 germline mutations in Chinese women with familial breast cancer.Breast Cancer Res Treat 132:421-428.

[27] Théry JC, Krieger S, Gaildrat P, Révillion F, Buisine MP,et al.(2011)Contribution of bioinformatics predictions and functional splicing assays to the interpretation of unclassified variants of the BRCA genes. Eur J Hum Genet 19:1052-1058.

[28] Michils G, Hollants S, Dehaspe L, Van Houdt J, Bidet Y,et al. (2012)Molecular analysis of the breast cancer genes BRCA1 and BRCA2 using amplicon-based massive parallel pyrosequencing.J Mol Diagn 14:623-630.

[29] Lecarpentier J, Noguès C, Mouret-Fourme E, Gauthier-Villars M, Lasset C,et al.(2012)Variation in breast cancer risk associated with factors related to pregnancies according to truncating mutation location, in the French National BRCA1 and BRCA2 mutations carrier cohort (GENEPSO).Breast Cancer Res.14:R99.

[30] Popp H, Kalb R, Fischer A, Lobitz S, Kokemohr I,et al.Cytogenet Genome Res. 2003;103:54-57.

Screening Fanconi anemia lymphoid cell lines of non-A, C, D2, E, F, G subtypes for defects in BRCA2/FANCD1.

[31] Esteban Cardeñosa E, Bolufer Gilabert P, Palanca Suela S, Oltra Soler S, Barragán González E,et al.(2008) Twenty-three novel BRCA1 and BRCA2 sequence alterations in breast and/or ovarian cancer families of Eastern Spain.Breast Cancer Res Treat112:69-73.

[32] Suriano G, Oliveira C, Ferreira P, Machado JC, Bordin MC,et al. (2003) Identification of CDH1 germline missense mutations associated with functional inactivation of the E-cadherin protein in young gastric cancer probands. Hum Mol Genet 12:575-582.

[33] Fidalgo P, Almeida MR, West S, Gaspar C, Maia L,et al. (2000) Detection of mutations in mismatch repair genes in Portuguese families with hereditary non-polyposis colorectal cancer (HNPCC) by a multi-method approach.Eur J Hum Genet 8:49-53.

[34] Hu F, Li D, Wang Y, Yao X, Zhang W,et al. (2013)Novel DNA variants and mutation frequencies of hMLH1 and hMSH2 genes in colorectal cancer in the Northeast China population. PLoS One 8:e60233.

[35] Auclair J, Busine MP, Navarro C, Ruano E, Montmain G,et al. (2006) Systematic mRNA analysis for the effect of MLH1 and MSH2 missense and silent mutations on aberrant splicing. Hum Mutat 27:145-154.

[36] Mueller-Koch Y, Vogelsang H, Kopp R, Lohse P, Keller G,et al. (2005) Hereditary non-polyposis colorectal cancer: clinical and molecular evidence for a new entity of hereditary colorectal cancer. Gut 54:1733-1740.

[37] Yuan Y, Zheng S.(1999)Mutations of hMLH1 and hMSH2 genes in suspected hereditary nonpolyposis colorectal cancer. Zhonghua Yi Xue Za Zhi 79:346-348.

[38] Mangold E, Pagenstecher C, Friedl W, Mathiak M, Buettner R,et al.(2005) Spectrum and frequencies of mutations in MSH2 and MLH1 identified in 1,721 German families suspected of hereditary nonpolyposis colorectal cancer.Int J Cancer 116:692-702.

[39] Vahteristo P, Tamminen A, Karvinen P, Eerola H, Eklund C,et al.(2001)p53, CHK2, and CHK1 genes in Finnish families with Li-Fraumeni syndrome: further evidence of CHK2 in inherited cancer predisposition.Cancer Res 61:5718-5722.

[40] Giráldez MD, Balaguer F, Bujanda L, Cuatrecasas M, Muñoz J,et al. (2010)MSH6 and MUTYH deficiency is a frequent event in early-onset colorectal cancer.Clin Cancer Res 16:5402-5413.

[41] Pérez-Cabornero L, Infante M, Velasco E, Lastra E, Miner C,et al.(2013)Evaluating the effect of unclassified variants identified in MMR genes using phenotypic features, bioinformatics prediction, and RNA assays.J Mol Diagn 15:380-390.

[42] Kariola R, Otway R, Lönnqvist KE, Raevaara TE, Macrae F,et al.(2003) Two mismatch repair gene mutations found in a colon cancer patient--which one is pathogenic?Hum Genet 112:105-109.

[43] Nielsen M, Franken PF, Reinards TH, Weiss MM, Wagner A,et al.(2005)Multiplicity in polyp count and extracolonic manifestations in 40 Dutch patients with MYH associated polyposis coli (MAP).J Med Genet 42:e54.

[44] McBride KL, Varga EA, Pastore MT, Prior TW, Manickam K,et al.(2010)Confirmation study of PTEN mutations among individuals with autism or developmental delays/mental retardation and macrocephaly.Autism Res 3:137-141.

[45] Liaw D, Marsh DJ, Li J, Dahia PL, Wang SI,et al.(1997) Germline mutations of the PTEN gene in Cowden disease, an inherited breast and thyroid cancer syndrome.Nat Genet16:64-67.

[46] Gallione C, Aylsworth AS, Beis J, Berk T, Bernhardt B,et al.(2010) Overlapping spectra of SMAD4 mutations in juvenile polyposis (JP) and JP-HHT syndrome. Am J Med Genet A 152A:333-339.

[47] Kim IJ, Ku JL, Yoon KA, Heo SC, Jeong SY,et al. Germline mutations of the dpc4 gene in Korean juvenile polyposis patients. Int J Cancer 86:529-532.

[48] Westerman AM, Entius MM, Boor PP, Koole R, de Baar E,et al.(1999)Novel mutations in the LKB1/STK11 gene in Dutch Peutz-Jeghers families.Hum Mutat 13:476-481.

[49] Yoon KA, Ku JL, Choi HS, Heo SC, Jeong SY,et al.(2000) Germline mutations of the STK11 gene in Korean Peutz-Jeghers syndrome patients.Br J Cancer 82:1403-1406.

[50] Lim W, Hearle N, Shah B, Murday V, Hodgson SV,et al. (2003) Further observations on LKB1/STK11 status and cancer risk in Peutz-Jeghers syndrome.Br J Cancer 89:308-313.
