# Supplementary material for: Clinicopathologic and Molecular Features of Colorectal Adenocarcinoma with Signet-Ring Cell Component
Source: PLoS One. 2016 Jun 14;11(6):e0156659. doi: 10.1371/journal.pone.0156659 (PMC4907485; doi:10.1371/journal.pone.0156659)
Supplement: S2 Table — N = the number of patients. (DOCX) [file pone.0156659.s005.docx]

S2 Table .Multigene Mutation Profiling by NGS

|  | Group A | Group B |
| --- | --- | --- |
| **N*** | 33 | 21 |
| **mutated N** | 27(81.8) | 16（76.2） |
| **APC** | 12（36.4） | 5（23.8） |
| **BMPR1A** | 5（15.2） | 3(14.3) |
| **BRCA1** | 9(27.3) | 5(23.8) |
| **BRCA2** | 7(21.2) | 2(9.5) |
| **CDH1** | 7(21.2) | 6(28.6) |
| **EPCAM** | 0(0) | 0(0) |
| **MLH1** | 4(12.1) | 5(23.8) |
| **MSH2** | 4(12.1) | 3(14.3) |
| **MSH6** | 7(21.2) | 4(19.0) |
| **PMS2** | 1(3.0) | 1(4.8) |
| **MUYTH** | 2(6.1) | 3(14.3) |
| **PTEN** | 2（6.1） | 2（9.5） |
| **SMAD4** | 12（36.4） | 10（47.6） |
| **STK11** | 4（12.1） | 3（14.3） |

*N= the number of patients
